# BAP1 dependent expression of long non-coding RNA NEAT-1 contributes to sensitivity to gemcitabine in cholangiocarcinoma

**DOI:** 10.1186/s12943-017-0587-x

**Published:** 2017-01-25

**Authors:** Mansi Parasramka, Irene K. Yan, Xue Wang, Phuong Nguyen, Akiko Matsuda, Sayantan Maji, Catherine Foye, Yan Asmann, Tushar Patel

**Affiliations:** 1 Department of Transplantation and Department of Cancer Biology, Mayo Clinic, 4500 San Pablo Road, Jacksonville, Florida 32224 USA; 2 Department of Health Sciences Research, Mayo Clinic, Jacksonville, FL USA

**Keywords:** Cholangiocarcinoma, LncRNA, BAP1, NEAT-1, Gemcitabine, Cisplatin, Combination therapy

## Abstract

**Background:**

Genetic alterations in chromatin modulators such as BRCA-1 associated protein-1 (BAP1) are the most frequent genetic alteration in intrahepatic cholangiocarcinomas (CCA). We evaluated the contribution of BAP1 expression on tumor cell behavior and therapeutic sensitivity to identify rationale therapeutic strategies.

**Methods:**

The impact of BAP1 expression on sensitivity to therapeutic agents was evaluated in CCA cells with a 7-fold difference in BAP1 expression (KMBC-low, HuCCT1-high) and genetically engineered haplo-insufficient BAP1 knockout cells. We also identified long non-coding RNA genes associated with loss of BAP1 and their role in therapeutic sensitivity.

**Results:**

Sensitivity to gemcitabine was greater in low BAP1 expressing or BAP1 knockout cells compared with the high BAP1 expressing cells or control haplo-insufficient cells respectively. Similar results were observed with TSA, olaparib, b-AP15 but not with GSK126. A differential synergistic effect was observed in combinations of gemcitabine with olaparib or GSK126 in KMBC cells and TSA or bAP15 in HuCCT1 cells, indicating BAP1 dependent target-specific synergism and sensitivity to gemcitabine. A BAP1 dependent alteration in expression of lncRNA NEAT-1 was identified by RT-PCR based lncRNA expression profiling, and an inverse relationship between this lncRNA and BAP1 was observed in analysis of the Tumor Cancer Genome Atlas cholangiocarcinoma dataset. Exogenous modulation of NEAT-1 and/or BAP1 expression altered tumor cell phenotype and modulated sensitivity to gemcitabine.

**Conclusions:**

NEAT-1 is a downstream effector of gemcitabine sensitivity in CCA. The expression of BAP1 is a determinant of sensitivity to therapeutic drugs that can be exploited to enhance responses through combination strategies.

**Electronic supplementary material:**

The online version of this article (doi:10.1186/s12943-017-0587-x) contains supplementary material, which is available to authorized users.

## Background

Cholangiocarcinoma (CCA) is a relatively rare malignancy in developed countries with 11,000 estimated new cases and 3,700 estimated deaths in the United States in 2016 [1]. CCA represents a heterogeneous group of tumors arising from biliary epithelial cells (or cholangiocytes) and associated with the intra- or extra-hepatic biliary tract. The disease has most often progressed to an advanced stage at the time of clinical presentation and recurrence rate is high despite surgery. Single-agent molecular targeted or systemic chemotherapy is the conventional approach for treatment of CCA but is not very effective. Therapeutic resistance can arise from inter-individual variation in sensitivity, and specificity of the target as well as the agent. The use of multimodality approaches that include combination therapies is a rational approach to improve therapeutic outcomes if based on knowledge of cellular mechanisms associated with CCA growth and drug response. Recent studies have identified alterations in chromatin modulators as the most frequent genetic alterations in intrahepatic cholangiocarcinoma (iCCA) [2, 3]. Amongst these, mutations in BRCA-1 associated protein-1 (BAP1) are the most common, occurring in 22-24% of cases; however, the clinical impact of mutation or loss of BAP1 in iCCA is yet unknown. The gene is located on chromosome 3p21 and encodes BAP1, a member of the ubiquitin C-terminal hydrolase superfamily of deubiquitinating enzymes that plays a critical role in chromatin remodeling together with ARID1A and PBRM1. Chromatin remodeling by BAP1 can involve interactions with several methylation and deacetylase components and result in modulation of gene expression. We have recently reported the involvement of long non-coding RNA (lncRNA) in therapeutic sensitivity in hepatocellular cancers, but their role in iCCA is unknown [4, 5]. Moreover, the mechanisms by which lncRNA expression is regulated in cancer cells remain very poorly understood. Thus, we sought to evaluate the effect of BAP1 on the modulation of lncRNA involved in therapeutic sensitivity in iCCA. Our studies identified a novel regulatory niche between BAP1 and lncRNA nuclear paraspeckle assembly transcript 1 (NEAT-1) in modulating CCA phenotype and chemotherapeutic responses to gemcitabine individually and in combination with target-specific agents such as EZH2 or PARP inhibitor, GSK126 or olaparib, respectively. Recent evidence suggests a critical role of NEAT-1 in tissue development and as a transcription regulator promoting oncogenic transcriptome and cell regulatory pathways [6–10]. BAP1 and NEAT-1 dictated exclusive drug sensitivity was observed across CCA cell lines suggesting a key mechanistic role in enhancing therapeutic effectiveness. These findings provide new insights into the role of tumor suppressor gene - BAP1 in regulating the expression of NEAT-1, a finding that could guide rationale combinatorial approaches to enhance therapeutic sensitivity in iCCA.

## Methods

### Cell lines, culture, and reagents

Human cholangiocarcinoma cell lines KMBC, HuCCT1, Mz-ChA-1, CCLP1, human non-malignant intrahepatic biliary cells H69, and human haploid cells with and without BAP1 knockout, HAP1 WT and HAP1 BAP1 KO cells (Horizon Genomics, Cambridge, UK) were obtained and cultured as follows: KMBC, HuCCT1, and CCLP1 cells were cultured in Dulbecco’s modified Eagle’s medium (DMEM) high-glucose medium (HyClone, Logan, UT), containing 10% fetal bovine serum (FBS) and 1% antibiotic–antimycotic (Life Technologies, Grand Island, NY). Mz-ChA-1 cells were cultured in CMRL 1066 media with 10% FBS, 1% L-glutamine and 1% antibiotic–antimycotic. H69 cells were cultured in hormonally supplemented DMEM/nutrient mixture F-12 Ham (GIBCO BRL, Gaithersburg, MD) (3:1) containing adenine, insulin, triiodothyronine-transferrin, hydrocortisone, epinephrine, epidermal growth factor, penicillin/streptomycin and 10% FBS. Haploid cells were cultured in IMDM media with 10% FBS and 1% antibiotic–antimycotic. All cell lines were subjected to STR analysis. Gemcitabine was obtained from Selleckchem (Houston,TX), cisplatin (Sigma-Aldrich, St. Louis, MO), olaparib and GSK126 were obtained from Selleckchem (Houston, TX) and Cayman chemical (Ann Arbor, MI) respectively; trichostatin A (Santa Cruz Biotechnology, Santa Cruz, CA), and b-AP15 (VLX1500) were provided by Dr. Asher Chanan-Khan. Compounds were dissolved in 100% cell culture-grade DMSO and diluted with culture medium to the desired concentration with a final DMSO concentration of 0.1-0.25%. DMSO 0.1-0.25% (v/v) was used as a solvent control.

### Spheroid cell growth assays

Multicellular tumor spheroids were generated by culturing cells in the AlgiMatrix 3D cell culture system (Grand Island, NY). 1 × 10^6^ KMBC cells were seeded in a 6-well plate in the medium described above using AlgiMatrix scaffold and 10% firming buffer and allowed to form spheroids at 37 °C in a 5% CO_2_ incubator for 7 days. After a 7 day incubation period, the scaffold was dissolved using 5 ml dissolving buffer and spheroids were harvested. For therapeutic response studies, spheroids were seeded (50 per well) in an ultra-low attachment 96-well plate, and viable spheroids counted at selected time points using Cell Titer Glo3D assay (Promega, Madison, WI).

### Transient transfections

siRNA against BAP1, NEAT-1 or non-targeting (NT) control sequences were obtained from Qiagen FlexiTube siRNA (Valencia, CA) (Additional file 1). Cells were transfected with either 100 nmol/L siRNA to BAP1 or 75 nmol/L siRNA to NEAT-1 or NT control using Lipofectamine 2000 (Life Technologies, Grand Island, NY) in Opti-MEM medium. Two different siRNA to each target gene were evaluated (sequences in Additional file 1), and the most efficacious one selected for further study. After 6 h, the medium was replaced with the respective complete culture medium containing 10% serum and the cells were incubated for 48 h before further study. BAP1 human cDNA clone, pCMV6-AC-BAP1 plasmid was purchased from Origene Technologies, Rockville, MD. KMBC cells were transfected using Lipofectamine 2000 (Life Technologies, Grand Island, NY) for 24 h with 2 μg BAP1 or pcDNA3.1 control vector before further study. BAP1 CRISPR/Cas 9 knockout plasmid and control CRISPR/Cas9 plasmid were obtained from Santa Cruz Biotechnology, Inc (Santa Cruz, CA). The plasmid was designed for maximum knockdown efficiency. Using Lipofectamine 2000 and plasmid transfection medium H69 cells were transfected with 1 μg of respective plasmid. After 48 h incubation the transfection efficiency was determined by fluorescent microscopy and cells were sorted by FACS analysis.

### Assessment of therapeutic drug effect on cell growth

Cells were counted using the Vi-CellXR Cell Viability Analyzer (Beckman Coulter, Brea, CA), seeded at 1 × 10^3^ cells/12 μl/well density in 384-well plate (Corning, NY) and incubated for at least 12 h prior to drug treatment. 200 μl, 2X of the highest drug concentration per drug was dispensed in quadruplets in a flat bottom 96-well plate (Corning, NY) followed by 100 μl of complete medium dispensed across rest of the plate. A BioTek Precision XS robot (Winooski, VT) was programmed to prepare serial dilutions across the plate. Cell viability was assessed after 72 h post-treatment using Cell Titer GloR 2.0 assay (Promega, Madison, WI) and a BioTek synergy HT- Plate Reader (Winooski, VT). Data was analyzed using GraphPad Prism to derive the EC50 or IC50 for each drug. For studies of drug interactions between gemcitabine and other drugs, cell viability was assessed after incubation with fixed ratio combinations based on individual drug IC50 values. Interactions were evaluated using a median effects analysis to derive a combination index (CI), with a CI < 1 indicating a synergistic interaction and CI > 1 suggesting an antagonistic effect.

### DNA sequencing

Genomic DNA was isolated using a spin-column based protocol (Qiagen, Valencia, CA). Sequencing primers for BAP1 gene were designed using GeneRunner (Additional file 2: Table S1). The extracted DNA was amplified by PCR using GoTaq Hot Start mix (Promega, Madison, WI) and purified using Exo-SAP (Affymetrix, Santa Clara, CA) to remove excess primers and dNTPs as per the manufacturer’s instructions. 1.6pM of forward or reverse primers and 5 μl of the purified PCR product was used for DNA sequencing. Electropherograms obtained were analyzed using SeqScape v2.5 (ABI, Applied Biosystems, Foster City, CA).

### RNA isolation and real-time PCR analysis

Total RNA was extracted from cells using TRIzol (Life Technologies) and treated with RNase-free DNase I (Qiagen, Valencia, CA). RNA concentration was measured using NanoDrop ND-2000 (Nano-Drop Technologies, Wilmington, DE). One microgram of RNA was reverse-transcribed to cDNA using iScript cDNA Synthesis Kit (BIO-RAD Laboratories, Inc., Hercules, CA), and real-time quantitative RT-PCR was performed using a LightCycler 96 System (Roche Diagnostics, Mannheim, Germany) to detect BAP1, NEAT-1, NDM29, MER11C, SNHG4, β-actin, and U6 using SYBR green I (SYBR® Advantage® qPCR Premix, Clontech, Mountain View, CA). RT-PCR primer sequences are listed in Additional file 2: Table S2. PCR based expression profiling of lncRNA was performed using the LncProfiler qPCR Array Kit (System Biosciences, Mountain View, CA), according to the manufacturer’s instructions. RNA from BAP1 knock-down/out cells were treated with DNase I and 2 μg of DNase-treated RNA was reverse transcribed. Real-time PCR was performed (2X Maxima SYBR Green with Rox) and the cycle number at which the reaction crossed a threshold (CT) was determined for each gene. Raw CT values were normalized using a median CT value (ΔCT = CT_lncRNA_- CT_median_). For each lncRNA, the relative amount of each lncRNA between 2 sample sets A and B was described using the equation 2^-ΔΔCT^, where ΔΔCT = ΔCT_A_ -ΔCT_B_.

### Western blot analysis

Protein lysates were obtained from cells grown in 100-mm dishes. Nuclear and cytoplasmic fractions were obtained using the NE-PER extraction kit (Pierce, Rockford, IL), Protease Inhibitor Cocktail Tablet and PhosSTOP Phosphatase inhibitor (Roche, Indianapolis, IN) according to the manufacturer’s instructions. Protein concentrations in fractions were determined using the BCA Protein Assay (Pierce, Rockford, IL). Equivalent amounts of protein were mixed with 4X LDS sample buffer, incubated for 10 mins at 70 °C and resolved by electrophoresis in a 4–12% Bis-Tris gel (Life-Tech) followed by dry transfer to nitrocellulose membranes using iBlot Dry Blotting system (Life-Tech). After blocking (Odyssey Blocking buffer), membranes were incubated with respective primary antibodies [BAP1 (1:250) or PCNA (1:1000) (both from Santa Cruz Biotechnology, Santa Cruz, CA) diluted in Odyssey blocking buffer solution and incubated at 4 °C overnight, followed by infrared dye-labeled secondary antibodies (1:10000). The protein of interest was detected using the LI-COR Odyssey infrared imaging system (LI-COR Bioscience, Lincoln, NE). Relative expression was determined by probing against loading control.

### Cell cycle analysis

Cells were plated at ~1.25 × 10^5^ cells/well in a 6-well plate and incubated at 37 °C for 12 h followed by 6 h incubation in serum-free medium. The medium was replaced with complete medium containing either IC50 concentration of GSK126 or gemcitabine or a combination or DMSO control. After 24 h, cells were pelleted by centrifugation at 300 *g* for 5 mins, fixed using cold 70% ethanol (KMBC cells) or 4% paraformaldehyde (HuCCT1 cells) for 15–30 mins and washed twice with PBS. The cells were re-suspended in PBS and incubated with 4 mg/ml RNase for 15 mins, and then re-suspended in PBS and incubated with 10 mg/ml Propidium Iodide (PI) for up to 30 mins.100 μl of cell solution was then transferred to a 96-well plate, analyzed using an Acea Novocyte flow cytometer, and cell cycle analysis was performed using the integrated software.

### Invasion assay

5 × 10^4^ cells were suspended in 200 μl serum-free medium and loaded onto the upper compartment of Transwell (Corning, Lowell, MA) 24-well plates with a pore size of 8.0 μm. Serum-free medium (500 μl) was added to the bottom. After 24 h, cells that had migrated through the membrane were fixed and stained using Diff-Quik (Astral Diagnostics, West Deptford, NJ). Migrated cells were identified and quantitated using a microscope and average counts from 5 or more fields of cells were obtained for each group.

### Anchorage independent growth assay

Cells transfected with siRNA to NEAT-1 or to respective nontarget control were seeded in 24-well plate in complete medium supplemented with 20% serum. Cells were grown in soft agar as described previously [11]. The final concentration of the bottom and top feeder layers of the agar system was 1.2% and the cell suspension layer was 0.8%. Cells were incubated for 7 days in a humidified incubator at 37 °C. The total number of colonies was quantified as a direct proportion of fluorescence. Alamar Blue (Biosource International, Camarillo, CA) was added to the wells, and fluorescence was measured using a BioTek synergy HT- Plate Reader (Winooski, VT) (excitation 530/25 nm; emission 580/50 nm).

### Analysis of lncRNA in human CCA

Raw sequences of 36 TCGA CCA RNAseq samples were obtained from TCGA website [12]. These samples were analyzed using a Mayo Clinic custom developed bioinformatics analysis pipeline which aligned the raw sequences to GRCh37 using TopHat 2.0 [13], counted the reads for known mRNAs and lncRNAs defined in ENSEMBL GTF file using featureCounts [14]. One outlier sample was detected by principle component analysis and removed from further analysis. Genes having zero read counts in all remaining samples were removed and the remaining genes were normalized by CQN method [15]. EdgeR R packages [16] was applied to compare 10 samples with highest *BAP1* gene expression to 10 samples with lowest *BAP1* gene expression, and differentially expressed genes were identified.

### Statistical analysis

Data were expressed as the mean ± standard deviation from at least three replicates, unless indicated otherwise. Comparisons between groups were performed using the two-tailed Student’s *t* test, one- or two-way ANOVA. Results were considered to be statistically significant when *P* < 0.05.

## Results

### Basal BAP1 expression in CCA cells

**Fig. 1:**
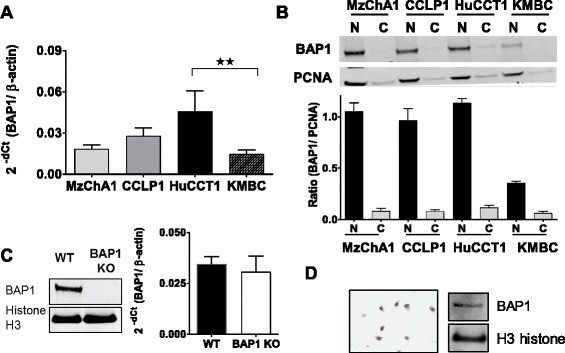
BAP1 protein and mRNA expression in CCA cells. **a**: BAP1 mRNA expression by quantitative RT-PCR (qRT-PCR). Data represents mean ± SEM of 2^-dCt^ values normalized to respective β-actin RNA (*n* = 3). **b**: Representative western blots and semi-quantitative analysis following normalization to PCNA of BAP1 protein expression in nuclear (N) or cytosolic (C) fractions under baseline conditions, from three independent studies. **c**: Western blot analysis of BAP1 protein expression in parental HAP1 cells (WT) and in HAP1 cells with CRISPR-Cas9 mediated knock-out of BAP-1 (BAP1 KO) The graph shows the mean ± SEM of 2^-dCt^ values from qRT-PCR expression analysis of BAP1 mRNA normalized to respective β-actin RNA (*n* = 3). * *P* < 0.05. **d**: BAP1 protein expression in H69 cells by immunocytochemistry and Western blot analysis in nuclear fraction of non-malignant H69 cells. Histone H3 was used as a reference control. BAP1 antibody was purchased from Santa Cruz (SC-28383) and used at a dilution of 1:250

In order to identify an appropriate cellular model, we began by first performing BAP1 gene mutation analysis by Sanger sequencing in a panel of human malignant cholangiocyte cell lines, KMBC, HuCCT1, Mz-ChA-1, and CCLP1. We identified several BAP1 mutations that spanned the entire genome (Additional file 2: Table S3). In KMBC and HuCCT1 cells, only a single point mutation within an intronic region was noted in each line, neither of which has been observed in iCCA in public databases such as cBioPortal, or SAGE. Next, we characterized BAP1 RNA and protein expression in all four cell lines. KMBC cells had the lowest, whereas HuCCT1 cells had the highest BAP1 mRNA expression (Fig. 1a). Similar results were observed with quantitative immunoblot analysis of BAP1 protein expression normalized to PCNA, with KMBC cells having low BAP1 protein expression, and HuCCT1 cells having the highest BAP1 protein (Fig. 1b). Based on these analyses, we selected KMBC cells and HuCCT1 cells as low- and high-BAP1 expressing cells for further analyses. Tumor suppressive effects resulting from BAP1 mutations likely involve additional genetic changes such as deletions of chromosome 3p. To evaluate the specific effect of loss of BAP1, we developed a complete BAP1 cellular knock-out model using HAP1 haploinsufficient cells. HAP1 cells are near haploid, and have only one set of chromosomes except for chromosome 8 and 15. A mutated phenotype is exposed immediately because there is only one copy of a gene on the haploid chromosomes. The use of these cells represents a powerful new technique to interrogate the phenotypic effects of genetic perturbations in human cancer cells, which is not possible with the use of diploid cells [17]. To examine the impact of specific loss of BAP1, BAP1 was knocked out in HAP1 cells using CRISPR/CAS9 (Additional file 2: Table S4). In these haplo-insufficient cells (designated as HAP1 BAP1 KO), there was a complete loss of BAP1 compared with the parental HAP1 WT cells (Fig. 1c).

### BAP1 modulates sensitivity to gemcitabine in CCA cells

**Fig. 2:**
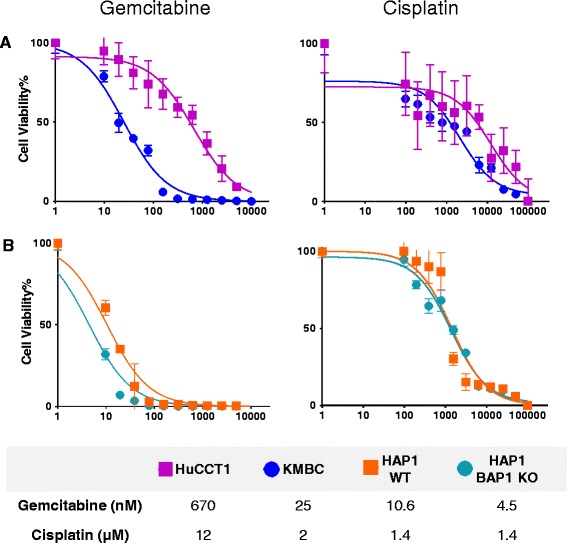
Concentration-dependent cytotoxic effect of gemcitabine or cisplatin. **a**: KMBC (blue circles) and HuCCT1 (purple squares) cells and **b**: haploid BAP1 knockout (HAP1 BAP1 KO) (*green circles*) or parental haploid HAP1 cells (WT) (*orange squares*) were plated in 384-well plates (1,000 cells/well), and incubated with varying concentrations of gemcitabine or cisplatin. Cell viability was assessed after 72 h using Cell Titer GloR 2.0 assay. Data represents the average percent cell viability plotted against concentration of drug in nM from 4 replicates for each condition. The table indicates the inhibitory concentration at 50% effect (IC50) values for each drug for each of the cell lines

We next evaluated the therapeutic sensitivity of CCA cells with low BAP1 (KMBC) or high BAP1 (HuCCT1) expression. Gemcitabine and Cisplatin are the current standard first line systemic agents for the treatment of CCA. Cells were incubated with varying concentrations of gemcitabine or cisplatin, for 72 h, and the inhibitory concentration at 50% effect (IC50) determined. We observed a selective drug response across cell lines (Fig. 2a). Sensitivity to gemcitabine was increased in low BAP1 expressing KMBC cells compared with high BAP1 expressing HuCCT1 cells (IC50: 25nM vs 670nM). Similar trends were observed upon treatment with cisplatin (IC50: 2 μM vs 12 μM respectively). To further validate the effect of BAP1 expression on drug sensitivity, we tested the therapeutic response of these drugs using a haploid cell BAP1 knockout model (Fig. 2b). HAP1 BAP1 KO cells or their respective control HAP1 WT cells were incubated with therapeutic agents, and concentration-effect curves analyzed. Sensitivity to gemcitabine was increased in HAP1 BAP1 KO cells, with an IC50 of 4.5nM compared with an IC50 of 10.6nM for control HAP1 WT cells. However these cells were insensitive to cisplatin, and there was no significant difference in sensitivity noted between the HAP1 BAP1 KO (IC50 of 1.4 μM) and HAP1 WT controls (IC50 of 1.4 μM). Taken together these studies indicate that BAP1 expression modulates sensitivity to gemcitabine.

### Evaluation of candidate therapeutics targeting BAP1-related drug resistance

**Fig. 3:**
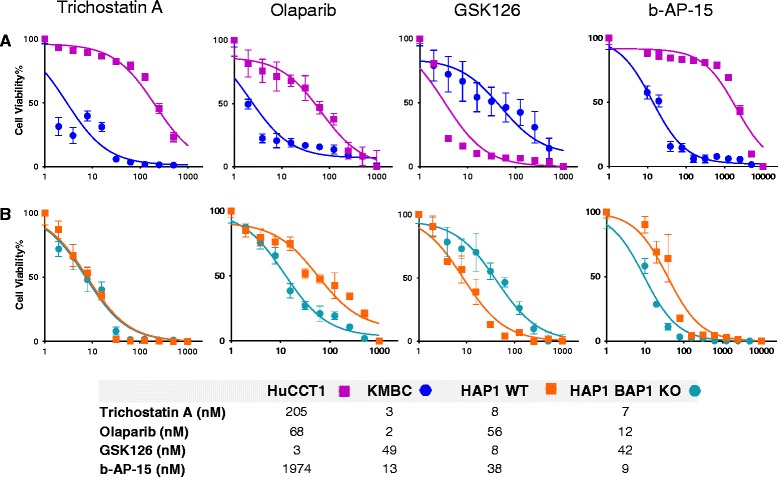
Concentration-dependent cytotoxic effect of candidate therapeutics. **a**. KMBC (blue circles) and HuCCT1 (purple squares) cells and **b**: haploid BAP1 knockout (HAP1 BAP1 KO) (*green circles*) or parental haploid HAP1 cells (WT) (*orange squares*) were plated in 384-well plates (1,000 cells/well), and incubated with varying concentrations of the indicated drug. Cell viability was assessed after 72 h using Cell Titer GloR 2.0 assay. Data represents the average percent cell viability plotted against concentration of drug in nM from 4 replicates for each condition. The table indicates the inhibitory concentration at 50% effect (IC50) values for each drug for each of the cell lines

We next evaluated whether therapeutic resistance related to BAP1 expression could be selectively targeted to improve therapeutic efficacy. To do so, we selected a panel of candidate compounds with the potential of modulating BAP1 function. The HDAC inhibitor trichostatin A (TSA) was selected based on the role of BAP1 in histone ubiquitination, the PARP inhibitor olaparib (Ola) was used to evaluate BAP1-related sensitivity to PARP inhibition, and b-AP15, an inhibitor of ubiquitin-specific-processing protease was selected to test against the deubiquitinating role of BAP1. Sensitivity to all of these therapeutic agents was increased in low BAP1 expressing KMBC cells compared with high BAP1 expressing HuCCT1 cells (Fig. 3a): TSA (IC50: 3nM vs 205nM), olaparib (IC50: 2nM vs 68nM), and b-AP15 (IC50: 13nM vs 1.9 μM). In contrast, sensitivity to GSK126, an inhibitor of enhancer of zeste 2 polycomb repressive complex 2 (EZH2) was reduced in KMBC compared with CCLP1 cells (IC50: 49nM vs 3nM). GSK126 can modulate anti-proliferative results of chromatin modification [18]. Similar trends were observed in haplo-insufficent BAP1 knock-out cells with IC50 values for HAP1 BAP1 KO cells compared to HAP1 WT cells as follows: TSA (7nM vs 8nM), Ola (12nM vs 56nM), b-AP-15 (9nM vs 38nM), GSK126 (42nM vs 8nM) (Fig. 3b). The R^2^ coefficient of determination values are provided in Additional file 2: Table S5. A greater sensitivity to EZH2 inhibition was noted in cells with higher BAP1 expressing cells (HAP1 WT, HuCCT1) than for low or absent BAP1 expressing cells (HAP1 BAP1 KO, KMBC). These results indicated that a, loss of BAP1 expression could modulate sensitivity to several drugs, and moreover that EZH2 inhibition could potentially be beneficial in high BAP1 expressing cells.

### Evaluation of combination therapies to enhance sensitivity to gemcitabine

**Fig. 4:**
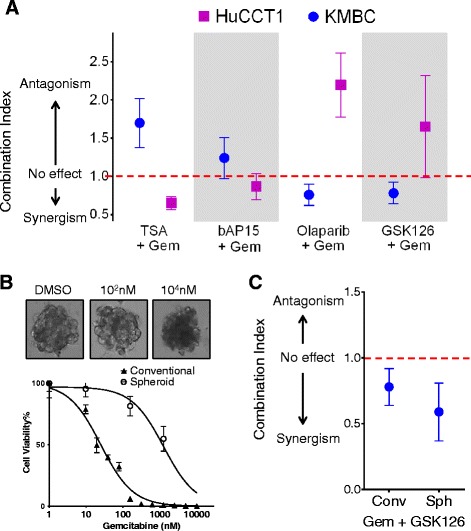
Synergistic interactions between candidate agents with gemcitabine. **a**: Cytotoxicity to drugs was assessed in KMBC (blue circles) and HuCCT1 (purple squares) cells incubated with gemcitabine (Gem) in combination with trichostatin A (TSA), b-AP15, olaparib, or GSK126 in a fixed ratio of concentrations, based on the IC50 of each drug. Potential interactions were evaluated using the median effects analysis of Chou and Talalay, and the combination index (CI) derived, with a CI < 1 indicative of a synergistic and a CI > 1 indicative of an antagonistic effect. At a fractional effect of 0.75, a synergistic effect was observed between Gem and TSA or b-AP15 in HuCCT1 cells but not with olaparib or GSK126, whereas the converse was noted in KMBC cells. **b**: KMBC cells grown in three-dimensional multicellular spheroids and incubated with gemcitabine. Representative morphological images in spheroid culture (top) and comparison of concentration dependent effects on cell viability cells in conventional in vitro or spheroid culture (bottom). The IC50 was 25nM in conventional and 1258nM in the spheroid cultures. **c**: Combination index from median effects analysis of combination treatment with gemcitabine and GSK126 in KMBC cells cultured under either conventional (conv) or multicellular spheroid culture. A synergistic effect was observed with cells in spheroid culture, similar to conventional (conv) in vitro culture conditions

To identify optimal combination strategies for high BAP1 expressing tumors, we next evaluated the effect of gemcitabine in combination with other candidate agents. A combination index was derived for interactions between gemcitabine and each of TSA, b-AP-15, olaparib and GSK126. Pharmacologic inhibition of EZH2 using GSK126 in combination with gemcitabine resulted in a synergistic reduction in cell viability in low BAP1 expressing cells, KMBC compared with high BAP1 expressing, HuCCT1 cells. At a fractional effect of 0.75, a synergistic effect occurred between gemcitabine and olaparib or GSK126 but not with TSA or b-AP-15 in KMBC cells (Fig. 4a). In marked contrast in HuCCT1 cells, a synergistic effect was not observed with either olaparib or GSK126, but was noted with TSA and b-AP-15. Moreover, differential effects on cell cycle progression were also observed with gemcitabine and GSK126 in KMBC and HuCCT1 cells. In the former, gemcitabine but not GSK126 arrested cell cycle progression, whereas in the latter GSK126 neither blocked progression nor enhanced the effect of gemcitabine (data not shown).

Next, we examined combination treatment with gemcitabine and GSK126 using an organotypic culture model system through the generation of a multicellular 3-dimensional (3D) KMBC spheroid system. Spheroid cultures can recapitulate native in vivo scenario and ensure an appropriate cell-to-cell contact environment that is typically presented in vivo. These 3D cultures provide an alternative to determine drug sensitivity that mitigates the inter-species differences in cell, microenvironment or drug metabolism associated with the use of other model organisms. KMBC cell-derived spheroids were developed as described in the methods section, with highly consistent size and number. Compared to 2D cell viability assays using KMBC cells, the IC50 dose for gemcitabine treatment was ~ 20-fold higher in 3D spheroid culture (IC50 25nM vs. 1258nM) (Fig. 4b). Multicellular KMBC spheroid cells treated with a combination of gemcitabine and GSK126 had a synergistic effect with a combination index value of 0.59 compared to CI = 0.78 for KMBC cells in conventional in vitro studies (Fig. 4c).

### BAP1 modulates expression of the lncRNA NEAT-1 in cholangiocytes

**Fig. 5:**
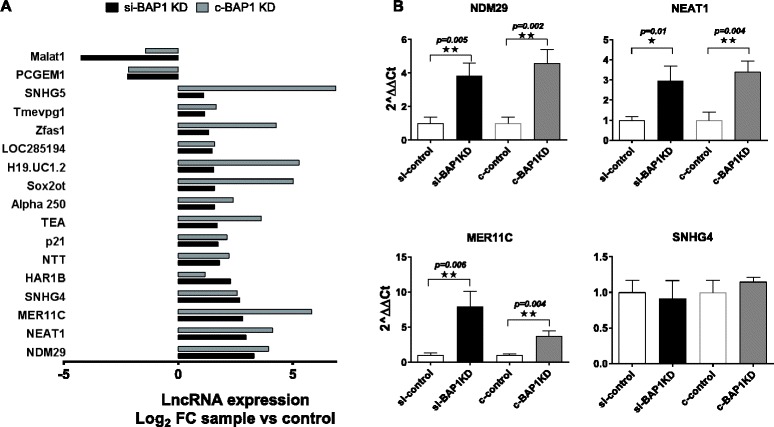
**a** Long non-coding RNA expression following modulation of BAP1 expression. LncRNA expression by qPCR was compared before and after experimental manipulation of BAP1 using either siRNA (si-BAP1 KD) or CRISPR/Cas9 in H69 cholangiocytes (c-BAP1-KD). **a**: Data indicates the average log_2_ fold-change (FC) in lncRNA expression in BAP1 knockdown cells from three independent replicates. **b** Expression of NDM29, NEAT-1, SNHG4, and MER11C was validated by qRT-PCR using different primer sets. Data represents the mean ± SEM of the ratio of lncRNA to RNU6B expression relative to controls*, *P* < 0.05

**Fig. 6:**
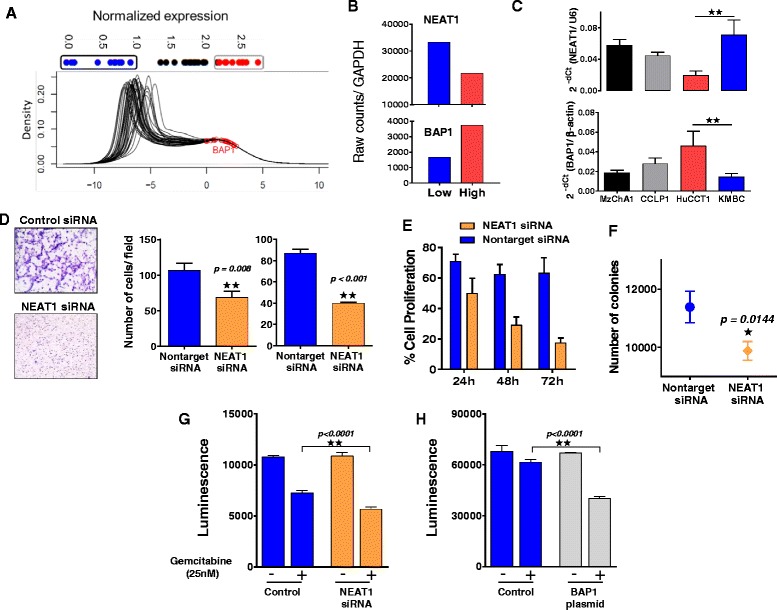
BAP1 and NEAT-1 in CCA. **a**: Density plot of the distribution of BAP1 expression across 35 iCCA samples from The Cancer Genome Atlas (TCGA) database, along with **b**: expression levels of NEAT-1 RNA in patients with low versus high BAP1 expression. **c**: BAP1 and NEAT-1 mRNA expression by qRT-PCR in human CCA cell lines. **d**: Migration and invasion assays were performed in H69 cells as described in the Methods section. Illustrative bright field microscopic images are shown (left), and number of migrating (middle) or invasive (right) cells/field. **e**. The percentage change in viable cell number assessed using Cell Titer GloR 2.0 assay at the indicated times following knockdown of NEAT-1. **f**. Anchorage-independent growth of NEAT-1-transfected cells was assessed using a soft agar assay, with fluorometric assessment of colony formation after 7 days. Bars represent the mean + SEM of 6 separate studies. *, *P* < 0.05. **g**, **h**: BAP1 and NEAT-1 were exogenously modulated in KMBC cells to determine sensitivity to gemcitabine. KMBC cells were plated in a 6-well culture plate and after 12 h, medium was replaced with serum-free medium and cells were transfected with (G) NEAT-1 or non-targeting control siRNA for 6 h, or (H) with BAP1 or control plasmid for 24 h. Cells were then harvested, re-seeded at equal density in a 384-well plate and incubated with 25nM gemcitabine. Cytotoxicity was determined using Cell Titer GloR 2.0 assay after 72 h. Bars represent the mean + SEM of 4 separate determinants. *, *P* < 0.05

The contribution of lncRNA to therapeutic responses is becoming recognized. We sought to identify BAP1 regulated candidate lncRNAs that could contribute to therapeutic sensitivity. BAP1 was knocked down in H69 non-malignant cholangiocytes using either siRNA to BAP1 or using a CRISPR-Cas9 BAP1 construct, and lncRNA expression was assessed using qRT-PCR based expression profiling. Suppression of BAP1 expression was confirmed by western and RT-PCR analysis. We identified several lncRNAs that were enriched in both siRNA and CRISPR-Cas9 mediated knock-down of BAP1 compared with the respective controls (Fig. 5a, Additional file 2: Table S6). Amongst these were four lncRNAs, NEAT-1, NDM29, MER11C, and SNHG4 that were at least 5-fold enriched with BAP1 knock-down with either system. Expression of these lncRNAs was further validated by qRT-PCR using independent primer sets, and we confirmed similar trends in the expression of NEAT-1, NDM29, and MER11C (Fig. 5b). NEAT-1 has two isoforms. Using qPCR and primer sets that target both NEAT1_1/NEAT1_2 (Total NEAT1), or NEAT1_1 alone, we observed an increase in both total NEAT-1 and NEAT1_1 with siRNA mediated modulation of BAP1 (Additional file 3). Moreover, the relative proportion of NEAT1_1 to total NEAT-1 was unchanged with BAP1 siRNA, suggesting that alteration in BAP1 equally affects both isoforms. For the rest of our studies, we studied total NEAT-1. We evaluated data from the TCGA (Fig. 6a), and observed an inverse correlation between BAP1 and NEAT-1 expression in iCCA samples consistent with observations made in the human CCA cell lines by qRT-PCR (Fig. 6b and c). Next, we evaluated the contribution of NEAT-1 on cancer cell phenotype. Exogenous modulation of NEAT-1 expression using siRNA against NEAT-1 (~50% knockdown) in KMBC cells reduced cell proliferation, migration, invasion and colony-forming abilities relative to non-targeting control siRNA treatment (Fig. 6d-f). These results indicate that NEAT-1 could contribute to the phenotypic effects associated with CCA.

### BAP1 and NEAT-1 regulate drug sensitivity in CCA cells

Given the effect of NEAT-1 on modulating the cancer cell phenotype, we evaluated the potential effect of NEAT-1 on sensitivity to gemcitabine. Modulation of NEAT-1 expression using siRNA in KMBC cells significantly altered their response to gemcitabine. At an IC50 concentration of 25nM gemcitabine, a significant reduction in NEAT-1 siRNA-treated cell proliferation was observed relative to non-target controls. The impact of BAP1 on sensitivity was further validated in studies in which KMBC cells were transfected with BAP1 expression plasmid, and noted to have increased cytotoxicity to gemcitabine compared with controls transfected with control plasmid transfected cells (Fig. 6g). Thus, NEAT-1 represents a functional downstream target of BAP1 involved in drug responses.

## Discussion

Alterations in chromatin modulators like the nuclear deubiquitinating enzyme BAP1 are the most frequently observed genetic alterations reported in intrahepatic CCA, yet the molecular mechanisms by which they modulate cancer cell behavior are unknown. BAP1 can act as a tumor suppressor and can regulate several cellular processes through its interaction with other protein partners such as host cell factor 1, O-linked N-acetylglucosamine transferase, transcription factor Ying Yang1, ASXL1/2, and FoxK1/K2 and DNA repair proteins like BRCA1/BARD1 heterodimer and RAD51. In the present study, we identify alterations in long non-coding RNA gene expression as a contributor to tumor cell phenotype and differential therapeutic sensitivity of CCA cells that is related to BAP1 expression.

Alterations in BAP1 expression in other cancers such as renal cell carcinoma, breast carcinoma, small cell and non-small cell lung cancers, malignant mesothelioma, metastasizing uveal melanoma, and hepatic cancers can arise from chromosomal deletions [19, 20]. Germline BAP1 mutations have been noted in several of these and other cancers such as meningioma, paraganglioma, basal cell carcinoma, ovarian cancers, and neuroendocrine tumors [21, 22]. Genetic mutations in BAP1 could modulate BAP1 protein expression or could alter BAP1 function in the absence of alterations in protein expression. Indeed, genetic mutations in BAP1 are variably associated with loss of protein expression. BAP1 protein expression has been assessed as a prognostic biomarker in iCCA. Loss of BAP1 expression by immunohistochemistry was noted in ~26% of a cohort of 211 patients with iCCA, and was associated with higher histological grade, absence of lymphatic invasion and a trend towards improved prognosis [3]. The greater sensitivity to gemcitabine and cisplatin in this setting may reflect favorable tumor biology and could contribute to these observations.

As a chromatin modulator, BAP1 is positioned to epigenetically modulate long non-coding RNAs (lncRNAs) and other genes at the intersection of cellular responses to an adverse microenvironment such as enhancement of survival signaling, modulation of cell proliferation and protein synthesis [23]. Our observations implicate a role for NEAT-1 as a downstream-target of BAP1 that is involved in responses to therapy as well as in maintenance of phenotypic characteristics such as the proliferative, migratory and invasive capabilities of CCA cells. The mechanisms by which NEAT-1 can modulate BAP1 effects are unknown. A role for NEAT-1 has been described in several types of cancers such as breast, prostate, and lung cancer, with effects on promoting tumor growth through genetic or epigenetic mechanisms [9, 10]. In pancreatic cancers, NEAT-1 can promote growth by modulating levels of miR-335-5p [8]. NEAT-1 can regulate gene expression through its participation in paraspeckle nuclear body formation, and further studies to explore how these effects may be related to modulation of therapeutic response are warranted. In addition to NEAT-1, several other lncRNAs have been implicated as modulators of drug sensitivity [24]. Examples include reports of lncRNA-HOTAIR in ER-induced tamoxifen resistance in breast cancer [25], lncRNA-UCA1 in cisplatin-based resistance in ovarian cancer cells [26], LncRNA-AK022798 in cisplatin-based drug resistance in gastric cancer cells [27] and H19 and lncRNA-ROR in hepatocellular carcinoma cells [4]. Consistent with the wide diversity of actions of lncRNA, several different mechanistic pathways have been implicated in these such as alterations in epithelial-mesenchymal transition, Wnt signaling, Notch 1, mitochondrial apoptosis, cell cycle regulation [28–30]. In another study, lncRNA ENST00000563280 and NR-036444 were found to interact with critical cancer genes such as ABCB1, HIF1A, and FOXC2 to modulate doxorubicin-resistance in osteosarcoma patients [31].

The dismal results observed with conventional therapies for CCA such as gemcitabine and cisplatin highlights the need for more effective drug regimens. Enhanced sensitivity to PARP inhibition has been reported in BAP1^−/−^ cells compared with BAP1^+/+^ and BAP1^+/−^ cells, as well as in cancer cells harboring inactivating mutations in BRCA1 or-2 [32, 33]. Our observations of increased sensitivity to PARP inhibition in the setting of reduced BAP1 expression are consistent with these reports and collectively support a role for BAP1 in DNA damage signaling and repair pathways that could promote the survival of cells with damaged DNA, drive neoplastic progression, as well as impact on response to drugs that induce DNA damage. Reduced BAP1 expression was also associated with enhanced sensitivity to bAP-15. An enhanced sensitivity to EZH2 inhibition was reported in mesothelioma cells lacking BAP1 [18], and related to enhanced expression of SETD8, a methyl transferase that decreased EZH2 expression and further restricted cell proliferation in these cells. In contrast to observations in other cancers, sensitivity to pharmacologic inhibition of EZH2 by GSK126 was decreased in the setting of reduced BAP1 expression. In CCA patients, EZH2 overexpression is associated with tumor stage, lymph node positivity, and poor prognosis [34]. Knockdown of EZH2 is reported to alter cell cycle regulation and induce G1 arrest leading to apoptosis in CCA cell lines [35]. We observed an increase in EZH2 protein and reduced SETD8 with siRNA knockdown of BAP1 confirming that EZH2 represents a potential therapeutic target that could be modulated by BAP1. Combination therapy of an EZH2 inhibitor, 3-deazaneplanocin A, and gemcitabine has had potent synergistic effects in CCA cells by causing cell cycle arrest and increased apoptosis [36].

## Conclusions

Knowledge of BAP1 expression could guide an appropriate selection of candidate drugs for combination approaches. Interestingly, both olaparib and GSK126 had synergistic interactions with gemcitabine in CCA cells, with reduced cell viability in low-BAP1 KMBC cells in contrast to the effect observed in high-BAP1 expressing HuCCT1 cells. The effect of NEAT-1 on modulation of EZH2 expression further supports an effect of this lncRNA on tumor growth. We speculate that low BAP1 expressing cells may have a higher expression of both lncRNA NEAT-1 and EZH2, with a lower sensitivity to EZH2 inhibition.

Overall, there are limited treatment options for CCA, a cancer that has been noted to be increasing in incidence in the US and many other countries. The heterogeneity of these cancers is being recognized. The evaluation of BAP1 expression, or even EZH2 expression, should be considered in future therapeutic studies as they may guide appropriate selection of synergistic combination approaches that could improve outcomes through improved therapeutic responses.

## Additional files

Additional file 1: siRNA modulation of BAP1 or NEAT-1. (PDF 70 kb)
Additional file 2: Supplementary Tables. (PDF 133 kb)
Additional file 3: Expression of NEAT-1 in malignant cholangiocytes. (PDF 11 kb)

## Abbreviations

BAP1: BRCA-1 associated protein-1
CCA: Cholangiocarcinoma
CI: Combination index
EZH2: Enhancer of zeste 2 polycomb repressive complex 2
IC50: Inhibitory concentration at 50% effect
lncRNA: Long non-coding RNA
NEAT-1: Nuclear paraspeckle assembly transcript 1
Ola: Olaparib
TSA: Trichostatin A
