# Supplementary material for: BAP1 dependent expression of long non-coding RNA NEAT-1 contributes to sensitivity to gemcitabine in cholangiocarcinoma
Source: Mol Cancer. 2017 Jan 25;16:22. doi: 10.1186/s12943-017-0587-x (PMC5264287; doi:10.1186/s12943-017-0587-x)
Supplement: Additional file 1: — siRNA modulation of BAP1 or NEAT-1. (PDF 70 kb) [file 12943_2017_587_MOESM1_ESM.pdf]

siRNA modulation of BAP1 or NEAT-1

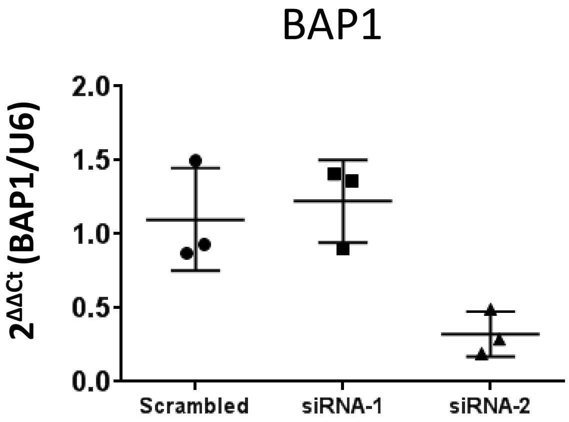

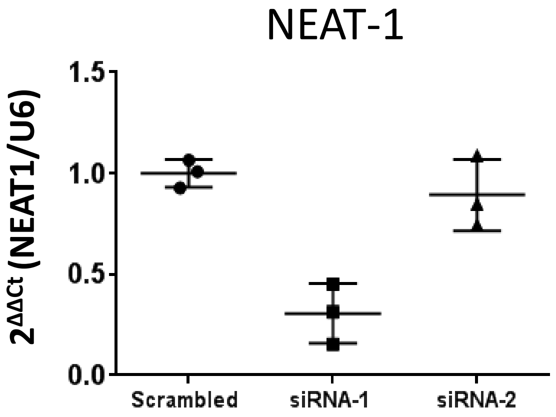

siRNA to BAP1
siRNA-1: CAGCAGCTGATAAGAGATAACA
siRNA-2: CTCAATTCCTCTGTCCATCAA

siRNA to NEAT1
siRNA-1: TTGCCTATCTAGTATCTTCAA
siRNA-2: GCCGGGAGGGCTAATCTTCAA
