# Supplementary material for: BAP1 dependent expression of long non-coding RNA NEAT-1 contributes to sensitivity to gemcitabine in cholangiocarcinoma
Source: Mol Cancer. 2017 Jan 25;16:22. doi: 10.1186/s12943-017-0587-x (PMC5264287; doi:10.1186/s12943-017-0587-x)
Supplement: Additional file 2: — Supplementary Tables. (PDF 133 kb) [file 12943_2017_587_MOESM2_ESM.pdf]

Additional file 2: Supplementary Tables

Expression of BAP1 modulates expression of long non-coding RNA NEAT-1 and contributes to sensitivity to gemcitabine in a BAP1-dependent manner in cholangiocarcinoma

Mansi Parasramka, PhD, Irene K. Yan, Xue Wang, PhD, Phuong Nguyen, Akiko Matsuda, MD, PhD, Sayantan Maji, PhD, Catherine Foye, Yan Asmann, PhD, Tushar Patel, MBChB

Table 1: BAP1 primer sequences for Sanger sequencing

Region	Primer sequence
**BAP1 ex123F**	GACTCTGTGTGTGGGACTGAG
**BAP1 ex123R**	AAGTATCAGTTTGATCAGGAG
**ex 4F**	TTATGCTTTGCTCTGCCTG
**ex 4R**	TATCTCCTCCTCCTGTCTTCC
**ex5F**	GACCAAAGATGAAAGGAGCTG
**ex5R**	ATGGTCTGACTTAAGGAAAC
**ex678F**	GACTCCACCCATAGTCCTACC
**ex678R**	GAACCCATGATCTAAGCC
**ex9F**	GACAGGTCTGCTGGTTCACTTC
**ex9R**	ATAATGGCCTTGGCTCTAC
**ex10F**	GACTTCCTTTAGGTCCTCAGC
**ex10R**	TTTAGGGAAGGACTGCTC
**ex11F**	TTTGCCCTGTCTCATTGC
**ex11R**	ACATTTAATCCCACAGCCAC
**ex12F**	GACCAAGGACAGGCCATGGAAC
**ex12R**	AACTCCGCAGGTGCTCAAC
**ex13F**	GACATGGCTAGTTCAAGTTGC
**ex13R**	AAATCATCAGAGTGCAGG
**ex14F**	GACTTTGGAGAGGATGACAGC
**ex14R**	AAGTCTTCTGGCACATGG
**ex1516F**	GACTTCTCCTTTAATCCTGGCAG
**ex1516R**	AGAGACCCTTGAGCAGGTG
**ex17F**	GACTGTCTTAACTGGAATGCC
**ex17R**	GAATGAAGAGAGAAGACCTG

Table 2: qPCR primer sequences

Identifier	Amplicon length	Forward primer	Reverse primer
**BAP1**, NG_031859	103	5’GCCTGAGGAGTCCAAGTCAG3’	5’CTGGAGGCTTCACCACTAGC3’
**Total NEAT1: NEAT1_1 (**NR_131012**) and NEAT1_2 (** NR_028272**)**	160	5’GACGAGATTAGATGGGCTCTTCTG3’	5’CGACCAAACACAGAAAAGACAACA3’
**NEAT1_1**, NR_131012	130	5′CAGTTAGTTTATCAGTTCTCCCATCCA3′	5′GTTGTTGTCGTCACCTTTCAACTCT3′
**NDM29**	98	5’GGCAGGCGGGTTGCTT3’	5’CCACGCCTGGCTAAGTTTTG3’
**SNHG4**, NR_003141	132	5’CCCATCTGAAGGGAGGAAATAG3’	5’GGGTCCTGATGGAGGTAGAT3’
**MER11C**	108	5’AAACTTGCTGATTTTGTGGCTT3’	5’TGTTGGCTGGTCTGTGAAATA3’
**U6**, NR_004394	83	5’CTCGCTTCGGCAGCACA3’	5’AACGCTTCACGAATTTGCGT3’
**Beta-actin** NC_000007.14	87	5’GGACTTCGAGCAAGAGATGG3’	5’ AGCACTGTGTTGGCGTACAG3’

Table 3: BAP1 mutation status in CCA cell lines

Cell line	Mutation type	Location	Position	Amino acid	Database
HuCCT1	Heterozygous	Intron 4	G>A		rs3821840
KMBC	Homozygous	Intron 8	A		
CCLP1	Heterozygous	Exon 15	1946 G>A	649 Cys>Tyr	rs151308667
MzChA1	Homozygous	Exon 1	31 G>A	11 Asp>Asn	
	Heterozygous	Exon 13	1583 C>A	528 Ser>Tyr	
	Homozygous	3'UTR	C>T		

Table 4: HAP1 BAP1 CRISPR/Cas9 Knockout

Forward Primer	Reverse Primer	Sequencing Primer
CAATATCATGTGGTAGCATTCCCAG	AACATTAGGCAAAGATGAAAGGAGC	AACATTAGGCAAAGATGAAAGGAGC

Sequencing Result

AGGATGAAGGCACTGCAGCCTACCTCAGGGCTGAAACCCCTGNTGAAGTCCTTCATGCGACTCAGGGTGGGTCCCAGGTCC
ACGCTGCTGCAGTTCAGGAGCACGCTCAAATCAATGGAGTTCCCGAAAGTCCATGCATTCTACTCTCACGACAATGGATTCA
TTCCAAAAGGAGCATCTTGTGGGAGCCGCCTTCCCTTTACAGGCATTCACCCCAAGCAAGGCATGAGTTGCACAAGAGTTGG
GTATCAGCTGTGAAACCAAGAATAGTCACCCATACACAGCACCCCTCACTGCAAGCCCCAAGCCCATATACATCAAGAATGG
GGATCCAGAGCAGGTCAGTCATATCTGGACAACTCCCCTTCTCAGCTCCTTTCATCTT

Table 5: R^2 value - Coefficient of Determination

Drug	HuCCT1	KMBC	HAP1 WT	HAP1 BAP1 KO
**Gemcitabine**	0.92	0.97	0.95	0.94
**Cisplatin**	0.60	0.87	0.92	0.97
**Trichostatin A**	0.95	0.81	0.95	0.96
**Olaparib**	0.91	0.84	0.91	0.97
**GSK126**	0.88	0.79	0.94	0.94
**b-AP-15**	0.91	0.95	0.92	0.95

Table 6: LncRNA expression profile in exogenously modulated BAP1 cells

LncRNA	si-BAP1 KD	c-BAP1-KD
MER11C	56.6	7.0
NDM29	15.3	9.9
NEAT-1	17.4	7.8
SNHG4	5.9	6.4
