# Supplementary figures and images for: BAP1 dependent expression of long non-coding RNA NEAT-1 contributes to sensitivity to gemcitabine in cholangiocarcinoma
Source: Mol Cancer. 2017 Jan 25;16:22. doi: 10.1186/s12943-017-0587-x (PMC5264287; doi:10.1186/s12943-017-0587-x)

## Expression of NEAT1 in CCA cells

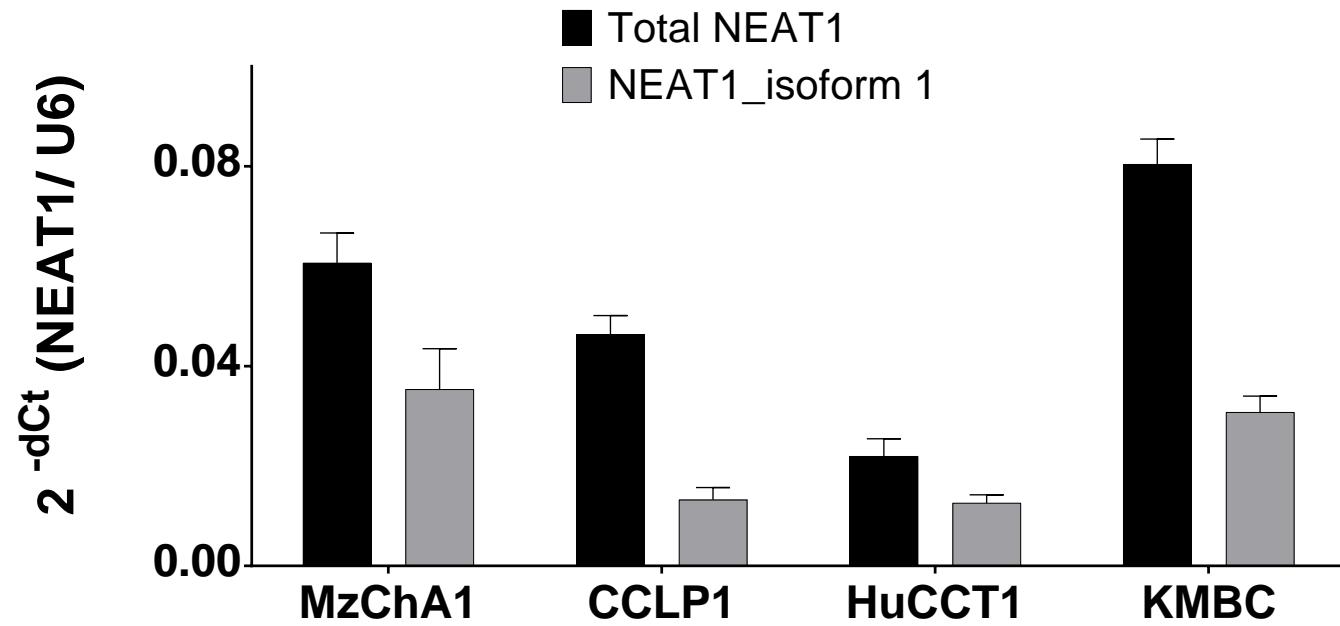

Supplement: Additional file 3: — Expression of NEAT-1 in malignant cholangiocytes. (PDF 11 kb) [file 12943_2017_587_MOESM3_ESM.pdf]
